# Distinct orbitofrontal circuits with dorsal and ventral CA1 differentially regulate spatial memory and emotional behaviors

**DOI:** 10.3389/fnbeh.2026.1853168

**Published:** 2026-06-10

**Authors:** Yanling Liang, Shiran Yu, Zizhuo Wang, Haibing Xu

**Affiliations:** Key Laboratory of Mental Health of the Ministry of Education, Guangdong-Hong Kong-Macao Greater Bay Area Center for Brain Science and Brain-Inspired Intelligence, Guangdong-Hong Kong Joint Laboratory for Psychiatric Disorders, Guangdong Province Key Laboratory of Psychiatric Disorders, Guangdong Basic Research Center of Excellence for Integrated Traditional and Western Medicine for Qingzhi Diseases, Department of Neurobiology, School of Basic Medical Sciences, Southern Medical University, Guangzhou, China

**Keywords:** dorsal CA1, emotion, orbitofrontal cortex, spatial memory, ventral CA1

## Abstract

**Background:**

The orbitofrontal cortex (OFC) and hippocampus are critically involved in cognitive and affective functions. However, the circuit architecture and functional organization linking the OFC with distinct hippocampal subregions, particularly dorsal CA1 (dCA1) and ventral CA1 (vCA1), remain poorly understood.

**Methods:**

We combined viral circuit tracing with a bilateral disconnection strategy to investigate the anatomical and functional organization of OFC–CA1 connectivity in mice. Dual viral tracing was used to map direct and polysynaptic pathways, and taCasp3-mediated targeted neuronal ablation was employed to assess circuit-specific functional contributions. Behavioral effects were evaluated using spatial memory assays (Y-maze and object location recognition) and emotion-related tests (open field, elevated plus maze, tail suspension, and forced swim tests).

**Results:**

We identified a unidirectional indirect pathway linking OFC to dCA1 via the claustrum (OFC → claustrum→dCA1), with no evidence of reciprocal dCA1 → OFC connectivity. In contrast, vCA1 exhibited a distinct connectivity profile, including a direct monosynaptic projection to OFC and multiple indirect pathways through the anterior thalamic nuclei, basolateral amygdala, and entorhinal cortex, while no OFC → vCA1 projection was detected. Functionally, disconnection of OFC–dCA1 connectivity selectively impaired spatial memory without affecting anxiety- or depression-like behaviors. Conversely, disconnection of vCA1–OFC connectivity induced anxiety- and depression-like phenotypes while preserving spatial memory.

**Conclusion:**

These findings demonstrate that the OFC interacts with dorsal and ventral CA1 subregions through anatomically distinct circuits with dissociable behavioral functions. This study provides circuit-level insight into how OFC–CA1 interactions differentially regulate spatial memory and affect-related behaviors.

## 1 Introduction

The orbitofrontal cortex (OFC) is a key prefrontal cortical region (Sequeira and Gourley, 2021) involved in reward processing, decision-making, and the regulation of emotional behaviors (Rolls, 2019a,b; Rolls et al., 2020; Zhang et al., 2024). Extensive evidence suggests that dysfunction of the OFC contributes to a variety of neuropsychiatric disorders, including depression, anxiety disorders, and cognitive impairments (Rolls, 2019b; Rolls et al., 2020; Knudsen and Wallis, 2022). In addition to its established role in value-based decision-making, the OFC integrates multimodal information, including sensory, emotional, and contextual inputs, to support flexible and adaptive behavior (Rolls, 2019b; Rolls et al., 2020). However, the circuit-level mechanisms through which the OFC coordinates distinct behavioral domains, particularly emotional and mnemonic processes, remain incompletely understood. Recent developmental neuroimaging studies further suggest that orbitofrontal-associated cortical network organization and morphometric patterns are linked to cognitive outcomes and vulnerability to internalizing symptoms, highlighting the translational relevance of defining specific OFC-related circuits involved in emotional and cognitive regulation (Yu et al., 2023; Kuang et al., 2023).

The hippocampus plays a central role in memory formation (O'Keefe and Dostrovsky, 1971) and emotional regulation (Pronier et al., 2023; Bannerman et al., 2014). Anatomically and functionally, the hippocampus exhibits a pronounced longitudinal specialization (Fanselow and Dong, 2010; Strange et al., 2014). The dorsal hippocampus is primarily involved in spatial learning and memory (O'Keefe and Dostrovsky, 1971; Bannerman et al., 2014), whereas the ventral hippocampus is more strongly associated with emotional processing (Bannerman et al., 2014). Within the hippocampal circuitry, the CA1 subregion serves as a major output structure that integrates information from upstream hippocampal areas and distributes it to multiple cortical and subcortical targets. Notably, the dorsal CA1 (dCA1) has been widely implicated in spatial navigation and working memory (Bannerman et al., 2014), while the ventral CA1 (vCA1) plays an important role in anxiety- and depression-related behaviors (Bannerman et al., 2014; Fanselow and Dong, 2010; Forro et al., 2022). This functional dissociation suggests that distinct upstream and downstream circuits may selectively engage dorsal or ventral hippocampal networks to regulate different behavioral domains.

Interactions between the OFC and hippocampus are thought to play an important role in integrating cognitive and emotional information (Rolls et al., 2022; Macoveanu et al., 2014; Knudsen and Wallis, 2020; Han et al., 2023). Previous studies have provided converging evidence for OFC–hippocampal interactions using multiple approaches, including diffusion tractography (Rolls et al., 2022), functional magnetic resonance imaging (Macoveanu et al., 2014), closed-loop theta stimulation (Knudsen and Wallis, 2020), and repetitive transcranial magnetic stimulation (Han et al., 2023). However, the precise anatomical organization of circuits linking the OFC with specific hippocampal subregions remains poorly defined. In particular, it is unclear whether the OFC forms distinct anatomical circuits with dCA1 and vCA1, and whether such circuits differentially contribute to spatial memory and emotional behaviors.

In the present study, we systematically investigated the anatomical and functional organization of OFC–hippocampal circuits using viral tracing and disconnection approaches. We found that the OFC forms distinct circuits with dorsal and ventral hippocampal CA1 subregions, which differentially regulate spatial memory and emotional behaviors. This work provides new insights into the circuit-level mechanisms underlying the functional specialization of hippocampal networks and highlights the role of OFC–hippocampal interactions in coordinating spatial memory and emotional processes.

## 2 Methods and materials

### 2.1 Animals

Laboratory mice were group-housed (4–5 per cage) in ventilated cages within a specific pathogen-free (SPF) animal room maintained at 22 ± 2 °C and 45 ± 10% humidity, under a 12-h light/dark cycle (lights on at 8:00, off at 20:00). Food and water were provided *ad libitum*. All animal procedures were performed in accordance with the ethical guidelines for animal experimentation and laboratory animal management regulations of Southern Medical University.

Male C57BL/6 mice were obtained from the Guangdong Provincial Medical Laboratory Animal Center. Ai9 mice were purchased from Jackson Laboratory (USA). The Ai9 strain is a Cre-dependent tdTomato reporter line on a C57BL/6 J genetic background, which exhibits strong red fluorescence following Cre-mediated recombination.

### 2.2 Viral neural circuit tracing

Ai9 reporter mice were anesthetized via intraperitoneal injection of 1.25% tribromoethanol solution (Avertin; 0.02 mL/g body weight) and head-fixed in a stereotaxic apparatus. The skull surface was leveled with reference to the bregma and lambda sutures. To map both direct and polysynaptic connections between the OFC and the hippocampal CA1 subregions, we employed a dual viral tracing strategy. A combination of retrograde (AAV2/retro-hSyn-EGFP) and anterograde transsynaptic (AAV2/1-hSyn-Cre) viruses was injected into targeted brain regions. Specifically, to trace dCA1 → OFC connections, AAV2/retro-EGFP was injected into the OFC and AAV2/1-Cre was injected into the dCA1. Conversely, to trace OFC → dCA1 connections, AAV2/retro-EGFP was injected into dCA1 and AAV2/1-Cre was injected into the OFC. For the vCA1 circuit mapping, parallel injections were performed: to trace vCA1 → OFC connections, AAV2/retro-EGFP was injected into OFC and AAV2/1-Cre into vCA1; and to trace OFC → vCA1 connections, AAV2/retro-EGFP was injected into vCA1 and AAV2/1-Cre into OFC. All viruses were injected at a rate of 1 nL/s. Stereotaxic coordinates relative to bregma (in mm) according to the Allen Mouse Brain Atlas were as follows: OFC (two injection sites per hemisphere: site 1: AP + 2.55, ML ± 0.50, DV -2.00; site 2: AP + 2.55, ML ± 1.50, DV -2.15); dCA1 (AP -1.65, ML ± 1.20, DV -1.52); vCA1 (AP -3.10, ML ± 3.06, DV -4.00). All viral injections were administered unilaterally within the same hemisphere. Following injections, the scalp was sutured. Mice were transcardially perfused three weeks post-injection for histological analysis.

### 2.3 Targeted neuronal ablation via taCasp3-TEVp

To investigate the functional role of the neural connectivity between OFC and dCA1/vCA1 (as mapped in Section 2.2) in spatial memory and emotional behaviors, we employed a bilateral disconnection strategy (Chudasama et al., 2012; Thonnard et al., 2021) to sever these neural pathways. Neuronal ablation was achieved using AAV2/9-hSyn-taCasp3-T2A-TEVp, a tool for inducing Caspase-3-dependent, targeted apoptosis (Yang et al., 2013). The taCasp3 construct contains the tobacco etch virus protease (TEVp) cleavage site inserted at the caspase-3 (Casp3) activation loop. Co-expression of TEVp leads to specific cleavage and irreversible activation of Casp3, initiating the apoptotic cascade in transduced neurons.

Two disconnection paradigms were used to assess OFC–dCA1 and OFC–vCA1 connectivity, respectively. For each pathway, mice were assigned to one of three groups: sham, ipsilateral ablation, or contralateral ablation. In the OFC–dCA1 disconnection experiment, unilateral OFC ablation was combined with either ipsilateral or contralateral dCA1 ablation. In the OFC–vCA1 disconnection experiment, unilateral OFC ablation was combined with either ipsilateral or contralateral vCA1 ablation. The contralateral ablation group served as the disconnection condition, whereas the ipsilateral ablation group served as the surgical control.

The same stereotaxic coordinates as in Section 2.2 were used. A mixture of AAV2/9-hSyn-taCasp3-T2A-TEVp and AAV2/9-hSyn-EGFP (1:1 titer ratio) was injected into the target brain regions designated for ablation. The co-injected AAV2/9-EGFP served as an internal histological control; successful neuronal ablation was confirmed by the loss of EGFP fluorescence in the target area. Sham control mice received injections of AAV2/9-hSyn-EGFP only.

Behavioral testing commenced 21 days post-injection to allow for maximal transgene expression and completion of apoptosis. Ablation efficacy was verified histologically upon completion of all behavioral tests.

### 2.4 Spatial memory behavioral paradigms

All spatial memory behavioral tests were conducted under controlled dim illumination conditions (~50 lux).

#### 2.4.1 Y-maze spontaneous alternation test

Spatial working memory was evaluated using the Y-maze spontaneous alternation test. Each mouse was placed at the end of one arm and allowed to explore freely for 8 min. Arm entries (all four paws within an arm) were recorded. An alternation was defined as consecutive entries into all three different arms. The percentage of spontaneous alternation was calculated as: Alternation (%) = [Number of alternations / (Total arm entries – 2)] × 100.

#### 2.4.2 Y-maze spatial novelty recognition test

This test comprised two 10-min phases separated by a 30-min interval. In the training phase, one arm was blocked, and mice explored the two accessible arms. In the test phase, all three arms were open. Exploration time traveled in each arm were recorded. Mice with intact spatial memory typically show increased exploration of the previously inaccessible (“novel”) arm.

#### 2.4.3 Object location recognition test (OLRT)

Spatial recognition memory was assessed using the OLRT. The apparatus was a 40 cm × 40 cm open field. During a 10-min training session, mice freely explored two identical objects. After a 30-min inter-trial interval in their home cages, mice underwent a 5-min test session where one object was moved to a novel location. Exploration was defined as directing the nose within <2 cm of the object. A preference index (PI) was calculated: PI = T_novel_/ (T_novel_ + T_familiar_), where T represents exploration time.

### 2.5 Emotion-related behavioral paradigms

All emotion-related behavioral tests were conducted under controlled dim illumination conditions (~50 lux).

#### 2.5.1 Open field test (OFT)

General locomotor activity and anxiety-like behavior were assessed in the open field. Mice were placed in the center of a square arena (40 × 40 × 40 cm) and allowed to explore freely for 10 min. Activity was tracked using an overhead camera and ANY-maze software. The arena was virtually divided into a central zone (20 × 20 cm) and a peripheral zone. Total distance traveled and time spent in the center zone were analyzed as measures of locomotor activity and anxiety-like behavior, respectively.

#### 2.5.2 Elevated plus maze (EPM)

Mice were acclimatized to the testing room for 1 h under dim light. Each mouse was placed in the center of the EPM, facing an open arm, and allowed to explore freely for 5 min. The time spent in and the number of entries into the open arms were recorded and analyzed.

#### 2.5.3 Tail suspension test (TST)

After 1-h acclimation, mice were suspended by the tail (2–3 mm from the tip) using adhesive tape on a hook, ~30 cm above the base. A 6-min session was video-recorded. Immobility time, defined as the absence of escape-oriented movements, was quantified.

#### 2.5.4 Forced swim test (FST)

Mice were placed individually in a clear cylinder (25 cm height × 20 cm diameter) filled to two-thirds with 25 ± 1 °C water for 6 min. Immobility was defined as passive floating with only minimal movements to keep the head above water. Mice were dried and returned to a warm cage after testing. Water was changed between trials.

### 2.6 Perfusion, tissue processing, and imaging

Three weeks after viral injections or upon completion of behavioral tests, mice were deeply anesthetized (intraperitoneal injection, 0.02 mL/g) and transcardially perfused. Perfusion was initiated with normal saline until the liver and kidneys cleared, followed by 4% paraformaldehyde (PFA) in PBS.

Brains were extracted, post-fixed in 4% PFA overnight at 4 °C, then cryoprotected in 30% sucrose solution until they sank. Coronal sections (40 μm thick) were cut on a cryostat and stored at −80 °C.

For imaging, sections were mounted and coverslipped. Whole-slide scanning was performed using a Nikon confocal microscope (10 × objective). Higher-resolution images were acquired using the same system with 20 × and 40 × objectives.

### 2.7 Statistical analysis

Statistical analyses were performed using GraphPad Prism (version 10.1.2). Data are presented as mean ± SEM. Normality and homogeneity of variance were assessed using the Shapiro–Wilk test and Levene’s test, respectively, prior to parametric testing. For comparisons among multiple groups, one-way ANOVA was used, followed by Tukey’s *post hoc* test for multiple comparisons. When data did not meet the assumptions of normality or homogeneity of variance, nonparametric tests (Kruskal–Wallis test followed by Dunn’s multiple comparisons test) were applied. All statistical tests were two-tailed, and a *p* value < 0.05 was considered statistically significant.

## 3 Results

### 3.1 Neural circuit connectivity between OFC and dCA1

**Figure 1:**
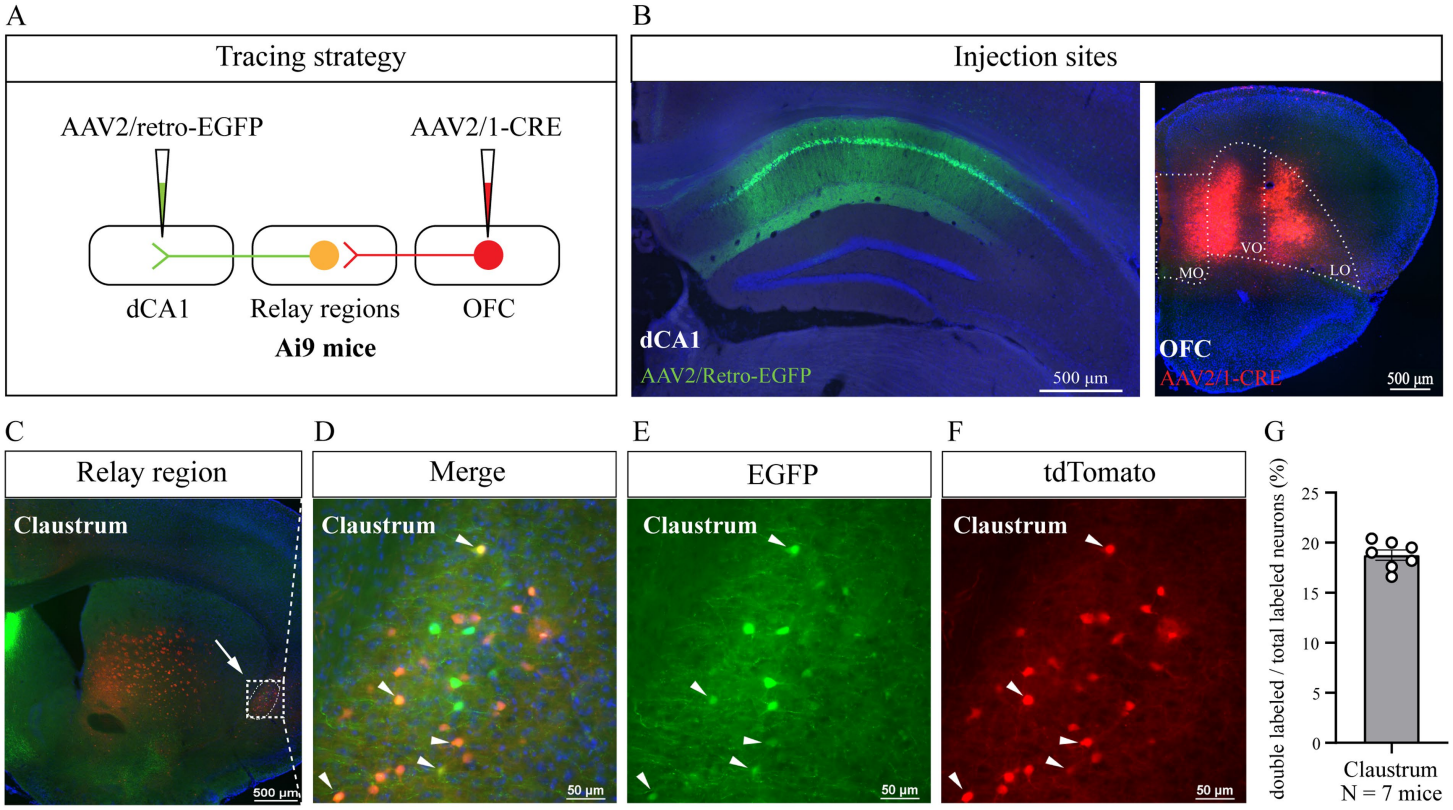
Identification of an indirect OFC→claustrum→dCA1 circuit. **(A)** Schematic illustration of the viral tracing strategy used to map connectivity between the OFC and dCA1 in Ai9 reporter mice. AAV2/retro-EGFP was injected into dCA1, and AAV2/1-Cre was injected into the OFC to identify intermediate relay regions linking OFC to dCA1. Cre-dependent tdTomato expression in Ai9 mice labeled neurons receiving transsynaptic input from OFC. Double-labeled neurons (EGFP^+^/tdTomato^+^) indicate relay cells within the pathway. **(B)** Representative images showing viral injection sites in dCA1 (left, green; AAV2/retro-EGFP) and OFC (right, red; AAV2/1-Cre). **(C)** Representative coronal section showing labeled neurons in the claustrum, identified as a relay region between OFC and dCA1. The boxed area indicates the region shown at higher magnification in **(D–F)**. **(D)** Merged high-magnification image of the claustrum showing EGFP^+^/tdTomato^+^ neurons. Arrowheads indicate double-labeled neurons. **(E)** EGFP channel showing retrogradely labeled neurons in the claustrum. **(F)** tdTomato channel showing transsynaptically labeled neurons in the claustrum. **(G)** Quantification of the proportion of double-labeled neurons among total labeled neurons in the claustrum. Data are presented as mean ± SEM; each dot represents one mouse (*n* = 7 mice).

We employed a dual viral tracing strategy to delineate the structural connectivity between OFC and dCA1, probing for both direct and indirect (polysynaptic) pathways. When a retrograde tracer (AAV2/retro-hSyn-EGFP) was injected into the OFC and an anterograde transsynaptic tracer (AAV2/1-hSyn-Cre) was injected into the dCA1 of Ai9 reporter mice, no evidence of a direct or indirect dCA1 → OFC projection was observed. Conversely, injection of AAV2/retro-EGFP into dCA1 and AAV2/1-Cre into OFC (Figures 1A,B) revealed a specific, indirect pathway. Fluorescent labeling was detected in the claustrum (Figures 1C–F), demonstrating a polysynaptic OFC → claustrum→dCA1 pathway confined to the same cerebral hemisphere. Quantification of double-labeled neurons within the claustrum is presented in Figure 1G. In summary, our tracing data demonstrate a unidirectional, disynaptic connection from OFC to dCA1, relayed via the claustrum. No evidence was found for a direct OFC → dCA1 projection, a direct dCA1 → OFC projection, or any other indirect dCA1-to-OFC pathway involving intermediate structures.

### 3.2 Neural circuit connectivity between OFC and vCA1

**Figure 2:**
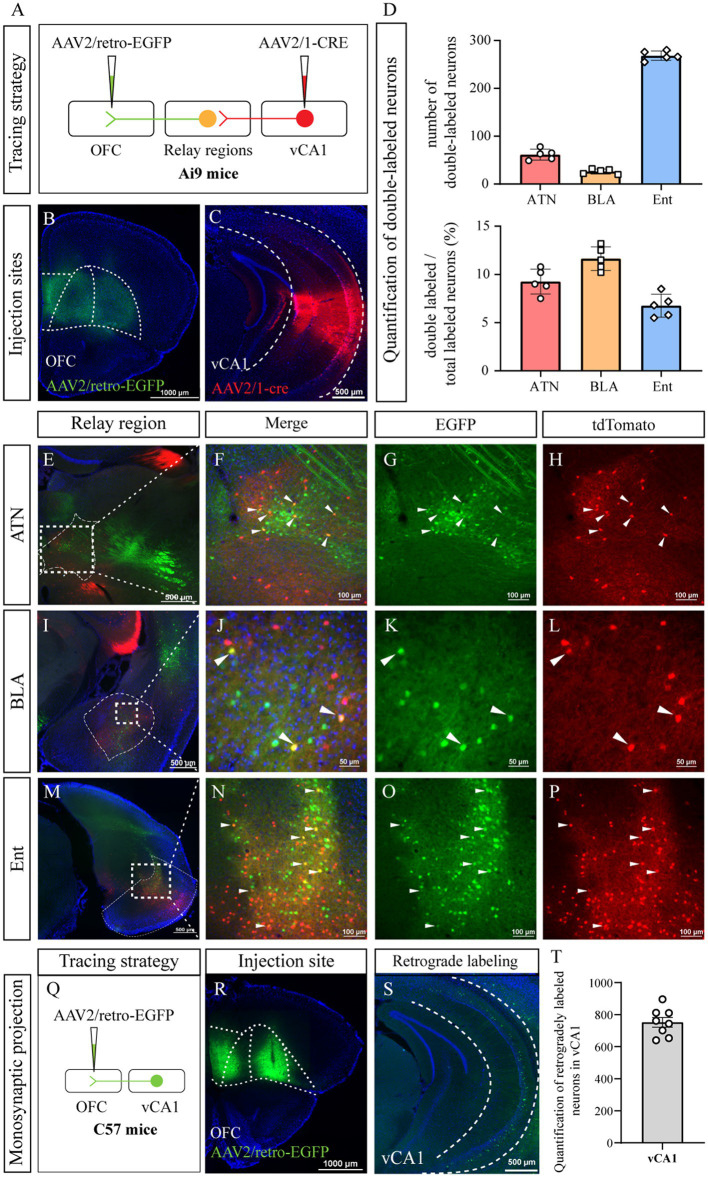
Identification of direct and indirect vCA1 → OFC circuits. **(A)** Schematic illustration of the viral tracing strategy used to identify relay regions linking vCA1 to OFC in Ai9 reporter mice. AAV2/retro-EGFP was injected into the OFC, and AAV2/1-Cre was injected into vCA1. Cre-dependent tdTomato expression in Ai9 mice labeled neurons receiving transsynaptic input from vCA1. Double-labeled neurons (EGFP+/tdTomato+) indicate relay cells within the vCA1 → OFC pathway. **(B,C)** Representative images showing viral injection sites in the OFC (**B**, green; AAV2/retro-EGFP) and vCA1 (**C**, red; AAV2/1-Cre). **(D)** Quantification of the number of double-labeled neurons (top) and the proportion of double-labeled neurons among total labeled neurons (bottom) in the ATN, BLA, and Ent. **(E,I,M)** Representative coronal sections showing labeled neurons in the ATN, BLA, and Ent, respectively, identified as relay regions between vCA1 and OFC. Boxed areas indicate the regions shown at higher magnification in **(F–H,J–L,N–P)**. **(F,J,N)** Merged high-magnification images of the ATN, BLA, and Ent showing EGFP+/tdTomato+ neurons. Arrowheads indicate double-labeled neurons. **(G,K,O)** EGFP channel showing retrogradely labeled neurons in the ATN, BLA, and Ent. **(H,L,P)** tdTomato channel showing transsynaptically labeled neurons in the ATN, BLA, and Ent. **(Q)** Schematic illustration of the tracing strategy used to verify the direct monosynaptic projection from vCA1 to OFC in C57 mice. AAV2/retro-EGFP was injected into the OFC. **(R)** Representative image showing the injection site of AAV2/retro-EGFP in the OFC. **(S)** Representative image showing retrogradely labeled neurons in vCA1, indicating a direct vCA1 → OFC projection. **(T)** Quantification of retrogradely labeled neurons in vCA1. Data are presented as mean ± SEM; each dot represents one mouse.

We next investigated the connectivity between the OFC and vCA1. Following injection of AAV2/retro-EGFP into vCA1 and AAV2/1-Cre into OFC, no evidence of a direct or indirect OFC → vCA1 projection was observed. However, the reciprocal tracing strategy (AAV2/retro-EGFP in OFC; AAV2/1-Cre in vCA1; Figures 2A–C,Q,R) revealed robust connectivity from vCA1 to the OFC. Specifically, we identified multiple indirect pathways mediated by distinct relay regions, including the anterior thalamic nuclei (ATN) (Figures 2E–H), the basolateral amygdala (BLA) (Figures 2I–L), and the entorhinal cortex (Ent) (Figures 2M–P), corresponding to the vCA1 → ATN → OFC, vCA1 → BLA → OFC, and vCA1 → Ent → OFC circuits, respectively. The proportion of double-labeled neurons within each of these relay nuclei (ATN, BLA, Ent) is quantified in Figure 2D. In addition, a direct monosynaptic projection from vCA1 to the OFC was observed (Figure 2S). Quantification of retrogradely labeled neurons in vCA1 is presented in Figure 2T.

### 3.3 The OFC–dCA1 connectivity selectively regulates spatial memory but not emotional behaviors

**Figure 3:**
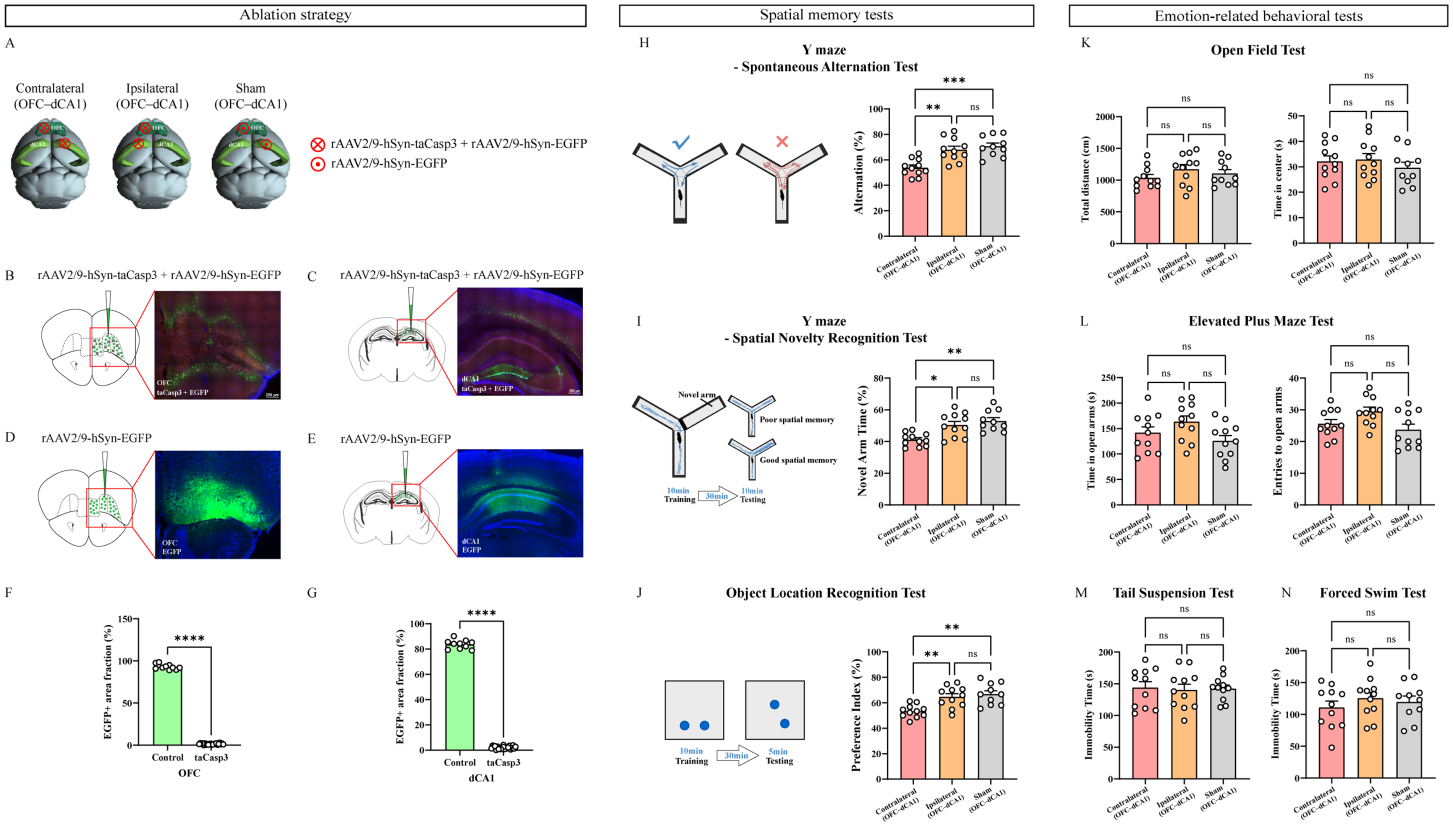
Disconnection of OFC–dCA1 connectivity selectively impairs spatial memory but not emotional behaviors. **(A)** Schematic illustration of the ablation strategy used to disrupt OFC–dCA1 connectivity. Mice were assigned to contralateral ablation (disconnection), ipsilateral ablation, or sham groups. Red crossed circles indicate injection of rAAV2/9-hSyn-taCasp3 + rAAV2/9-hSyn-EGFP; red open circles indicate injection of rAAV2/9-hSyn-EGFP only. **(B,C)** Representative fluorescence images showing marked loss of EGFP-positive neuronal labeling in the OFC **(B)** and dCA1 **(C)** following injection of rAAV2/9-hSyn-taCasp3 + rAAV2/9-hSyn-EGFP, indicating successful taCasp3-mediated neuronal ablation. **(D,E)** Representative images showing control viral expression in the OFC **(D)** and dCA1 **(E)** following injection of rAAV2/9-hSyn-EGFP alone. **(F,G)** Quantification of EGFP-positive area fraction within the targeted OFC **(F)** and dCA1 **(G)** regions following taCasp3-mediated neuronal ablation. **(H)** Y-maze spontaneous alternation test. Disconnection of OFC–dCA1 connectivity significantly reduced spontaneous alternation percentage. **(I)** Y-maze spatial novelty recognition test. Disconnection of OFC–dCA1 connectivity significantly reduced novel arm exploration time. **(J)** Object location recognition test. Disconnection of OFC–dCA1 connectivity significantly reduced the preference index. **(K)** Open field test. Disconnection of OFC–dCA1 connectivity did not significantly alter total distance traveled or time spent in the center. **(L)** Elevated plus maze test. No significant differences were observed in time spent in the open arms or number of entries into the open arms. **(M)** Tail suspension test. No significant difference in immobility time was detected among groups. **(N)** Forced swim test. No significant difference in immobility time was detected among groups. Data are presented as mean ± SEM; each dot represents one mouse. Statistical analyses for **(F,G)** were performed using unpaired Student’s *t* tests. Statistical analyses for **(H–N)** were performed using one-way ANOVA followed by Tukey’s multiple comparisons test. *p < 0.05; **p < 0.01; ***p < 0.001; ns, not significant.

To assess the functional role of the identified OFC-dCA1 connectivity, we performed bilateral disconnection lesions. Mice received contralateral (disconnection), ipsilateral, or sham (control) injections of the virus mix (AAV2/9-hSyn-taCasp3-TEVp + AAV2/9-hSyn-EGFP) or control virus (AAV2/9-hSyn-EGFP) into the OFC and dCA1 (Figure 3A). Histological verification confirmed successful ablation in the target regions (Figures 3B,C) and specific viral expression in control groups (Figures 3D,E). Quantitative analysis of EGFP-positive area fraction further demonstrated a significant reduction in EGFP-positive neuronal labeling within the targeted regions following taCasp3-mediated ablation compared with control animals (Figures 3F,G; unpaired Student’s *t* test, OFC: *t* = 128.80, df = 30, *p* < 0.0001; dCA1: *t* = 98.02, df = 30, *p* < 0.0001).

In spatial memory tests, the OFC-dCA1 disconnection group showed significant impairments compared to both the ipsilateral and sham control groups. One-way ANOVA revealed significant group effects on spontaneous alternation performance in the Y-maze [Figure 3H; *F* (2,29) = 13.60, *p* < 0.0001], novel arm exploration in the Y-maze spatial novelty recognition test [Figure 3I; *F* (2, 29) = 10.79, *p* = 0.0003], and preference index in the OLRT [Figure 3J; *F* (2, 29) = 10.35, *p* = 0.0004]. Tukey’s *post hoc* comparisons demonstrated significantly reduced performance in the contralateral disconnection group relative to both the ipsilateral and sham groups.

In contrast, the disconnection of the OFC-dCA1 connectivity did not affect emotional or locomotor behaviors. No significant differences were observed between groups in the OFT (center time, total distance) (Figure 3K), the EPM (open arm time, open arm entries) (Figure 3L), TST (immobility time) (Figure 3M), or the FST (immobility time) (Figure 3N) (one-way ANOVA, all *p* > 0.05). The unchanged total distance in the OFT confirmed that the observed spatial memory deficits were not attributable to alterations in general locomotor activity.

These results suggest that the OFC–dCA1 connectivity selectively contributes to spatial memory but not emotional behaviors.

### 3.4 The vCA1-OFC connectivity selectively regulates emotional behaviors but not spatial memory

**Figure 4:**
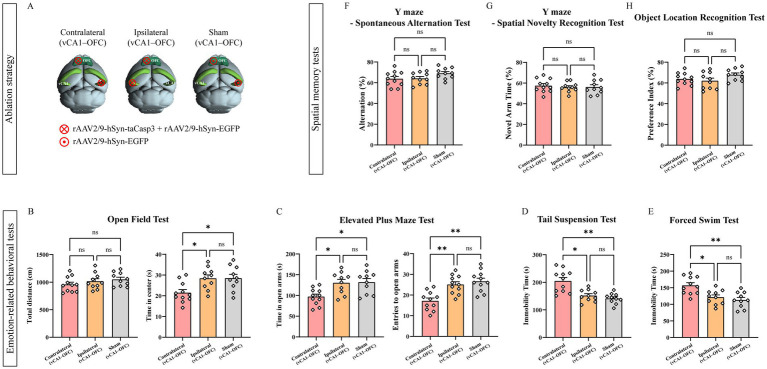
Disconnection of vCA1–OFC connectivity selectively alters emotional behaviors but spares spatial memory. **(A)** Schematic illustration of the ablation strategy used to disrupt vCA1–OFC connectivity. Mice were assigned to contralateral ablation (disconnection), ipsilateral ablation, or sham groups. Red crossed circles indicate injection of rAAV2/9-hSyn-taCasp3 + rAAV2/9-hSyn-EGFP; red open circles indicate injection of rAAV2/9-hSyn-EGFP only. **(B)** Open field test. Disconnection of vCA1–OFC connectivity did not significantly alter total distance traveled, but significantly reduced time spent in the center. **(C)** Elevated plus maze test. Disconnection of vCA1–OFC connectivity significantly reduced time spent in the open arms and the number of entries into the open arms. **(D)** Tail suspension test. Disconnection of vCA1–OFC connectivity significantly increased immobility time. **(E)** Forced swim test. Disconnection of vCA1–OFC connectivity significantly increased immobility time. **(F)** Y-maze spontaneous alternation test. No significant difference in spontaneous alternation percentage was observed among groups. **(G)** Y-maze spatial novelty recognition test. No significant difference in novel arm exploration time was observed among groups. **(H)** Object location recognition test. No significant difference in preference index was observed among groups. Data are presented as mean ± SEM; each dot represents one mouse. Statistical analyses were performed using one-way ANOVA followed by Tukey’s multiple comparisons test. *p < 0.05, **p < 0.01; ns, not significant.

We next examined the functional role of the vCA1-OFC connectivity using the same bilateral disconnection strategy (Figure 4A).

Disconnection of the vCA1-OFC connectivity induced an anxiety- and depression-like phenotype. One-way ANOVA revealed significant group effects on center time in the OFT [Figure 4B; *F* (2, 28) = 6.01, *p* = 0.0067], open-arm exploration in the EPM [Figure 4C; open-arm time: *F* (2, 28) = 6.87, *p* = 0.0037; open-arm entries: *F* (2, 28) = 11.66, *p* = 0.0002], TST immobility time [Figure 4D; *F* (2, 28) = 10.46, *p* = 0.0004], and FST immobility time [Figure 4E; *F* (2, 28) = 9.86, *p* = 0.0006]. Tukey’s *post hoc* comparisons demonstrated that the contralateral disconnection group exhibited significantly reduced center and open-arm exploration, as well as increased immobility time, compared with both the ipsilateral and sham control groups.

Conversely, spatial memory performance remained intact. No significant differences were observed between groups in Y-maze spontaneous alternation (Figure 4F), exploration of the novel arm in the Y-maze spatial novelty test (Figure 4G), or the preference index in the OLRT (Figure 4H) (one-way ANOVA, all *p* > 0.05). The lack of difference in total distance traveled in the OFT (Figure 4B) confirms that the emotional behavioral effects are independent of changes in basal locomotion.

These findings indicate that the vCA1-OFC connectivity selectively regulates emotional behaviors but not spatial memory.

## 4 Discussion

In the present study, we systematically characterized the anatomical and functional organization of OFC–hippocampal circuits linking the OFC with dorsal and ventral hippocampal CA1 subregions. Our principal findings are as follows. First, the OFC and dCA1 are connected through a unidirectional indirect pathway, in which OFC signals reach dCA1 via the claustrum, whereas no evidence was found for a reciprocal dCA1 → OFC projection. Second, the relationship between vCA1 and OFC is anatomically distinct: vCA1 sends both a direct monosynaptic projection to the OFC and several indirect polysynaptic projections through the ATN, BLA, and Ent. Third, these anatomically distinct circuits exhibit clear functional specialization. Bilateral disconnection of OFC–dCA1 connectivity selectively impaired spatial memory without affecting anxiety- or depression-like behaviors, whereas disconnection of vCA1–OFC connectivity selectively disrupted emotional behaviors while sparing spatial memory. Together, these findings reveal a circuit architecture through which the OFC interacts with longitudinally distinct hippocampal subnetworks to differentially regulate spatial memory and emotional behaviors.

Our tracing data extend current understanding of OFC–hippocampal communication by demonstrating that interactions between these structures are not organized as a single uniform pathway, but rather as topographically and directionally distinct subnetworks. Previous human studies have suggested functional coupling between the OFC and hippocampus across multiple contexts. For example, closed-loop theta stimulation studies have shown that hippocampal theta oscillations can drive OFC activity and are critical for reward-guided learning (Knudsen and Wallis, 2020). Functional MRI studies have further reported coordinated alterations in OFC–hippocampal activity during reward processing in individuals at risk for depression (Macoveanu et al., 2014), while neuromodulation studies using TMS-EEG have implicated OFC–hippocampal interactions in the modulation of depressive symptoms (Han et al., 2023). In addition, diffusion tractography studies have identified structural connectivity between the OFC and hippocampal system, supporting a role for this pathway in integrating reward information with memory and goal-directed behavior (Rolls et al., 2022). However, the precise anatomical substrates underlying these interactions have remained insufficiently resolved. By separately examining dCA1 and vCA1, our study shows that the OFC engages dorsal and ventral hippocampal systems through markedly different circuit motifs. This distinction is important because it provides an anatomical basis for the long-recognized functional heterogeneity along the hippocampal longitudinal axis.

One notable finding is the identification of a polysynaptic OFC → claustrum→dCA1 circuit. The claustrum is increasingly recognized as an integrative hub with widespread cortical connectivity and proposed roles in salience processing, attention, and multimodal integration (Smith et al., 2020; Madden et al., 2022; Zahacy et al., 2024; Jackson et al., 2020). Its position between OFC and dCA1 suggests that it may relay executive or contextual signals from OFC to dorsal hippocampal memory networks. One possible interpretation is that claustral processing may help coordinate OFC-derived task-related or contextual information with dorsal hippocampal-dependent spatial representations, thereby contributing to the flexible updating of spatial memory during behavioral exploration. Such an arrangement is consistent with the role of dCA1 in spatial representation and memory updating, and suggests that the OFC may influence spatial working memory not through direct anatomical control of hippocampal output neurons, but via intermediate associative structures that shape hippocampal information processing. The absence of a detectable direct OFC → dCA1 projection in our experiments supports the view that OFC modulation of dorsal hippocampal-dependent cognition may rely on distributed, multi-node circuits rather than simple monosynaptic communication.

Importantly, the present tracing strategy does not resolve the precise cellular identity of the claustral neurons participating in this pathway. Previous studies have suggested that long-range claustral projection neurons are predominantly glutamatergic excitatory neurons, whereas local inhibitory interneurons may contribute to information gating and synchronization within claustral microcircuits (Jackson et al., 2020). Although the current study does not directly distinguish these neuronal subpopulations, our findings raise the possibility that distinct excitatory and inhibitory claustral cell types may differentially contribute to OFC–dCA1 communication and signal integration. Future studies combining cell type-specific tracing, immunohistochemistry, or electrophysiological approaches will be necessary to determine the precise neuronal subtypes and synaptic mechanisms underlying this pathway.

Functionally, this anatomical organization was mirrored by the behavioral consequences of disconnection. Disrupting OFC–dCA1 connectivity impaired performance in spontaneous alternation, spatial novelty recognition, and object location recognition, indicating deficits in both spatial working memory and short-term spatial recognition memory. Importantly, these impairments occurred in the absence of changes in locomotor activity, anxiety-like behavior, or depression-like behavior, arguing against nonspecific effects of lesion burden or motivational disturbances. These findings support a model in which OFC input to dorsal hippocampal circuitry contributes specifically to spatial mnemonic processes. Although the OFC is classically associated with value representation and flexible decision-making (Rolls, 2019a,b; Knudsen and Wallis, 2022), growing evidence suggests that it also participates in cognitive map-like representations related to task structure and expected outcomes (Rolls et al., 2022; Wikenheiser et al., 2021; Yoo et al., 2018; Schuck et al., 2016). Our data raise the possibility that OFC-derived information may help dCA1 encode or retrieve spatial and context-related relationships relevant for adaptive behavior. Although the present study focused primarily on spatial memory and affect-related phenotypes, future studies incorporating reward-based or value-guided behavioral paradigms may help further determine whether anatomically distinct OFC–hippocampal circuits also contribute to reward processing and adaptive behavioral control.

In contrast to the dorsal pathway, the ventral hippocampal circuit exhibited a very different anatomical and functional profile. We found that vCA1 projects to OFC both directly and indirectly through the ATN, BLA, and Ent. This organization suggests that vCA1 may exert a more immediate and diversified influence on OFC processing. Given the established role of vCA1 in anxiety, stress responsivity, and affective regulation (Bannerman et al., 2014; Fanselow and Dong, 2010; Strange et al., 2014; Forro et al., 2022), such connectivity is well positioned to convey emotional and contextual state information to the OFC, which is involved in valuation and behavioral control. The presence of multiple relay nodes is also notable. The BLA is a key structure involved in emotionally salient and threat-related processing (O'Neill et al., 2018; Sun et al., 2020; Liu et al., 2025); the Ent provides an interface between hippocampal representations and cortical association networks (Schultz and Engelhardt, 2014; Garcia and Buffalo, 2020; Coutureau and Di Scala, 2009); and the ATN is increasingly recognized as part of extended hippocampal memory circuits (Hintiryan et al., 2025; Perry et al., 2021). These observations raise the possibility that the parallel vCA1–OFC relay pathways may contribute differently to the transfer of affective, contextual, or memory-related information, although the present study was not designed to functionally dissociate the specific contributions of individual relay nodes. Therefore, the vCA1 → OFC network may form part of a distributed network linking ventral hippocampal, limbic, and orbitofrontal regions, through which affective and contextual information may influence OFC processing.

Consistent with this interpretation, disconnection of the vCA1–OFC circuit produced robust anxiety- and depression-like phenotypes, as evidenced by reduced center exploration in the OFT, reduced open-arm exploration in the EPM, and increased immobility in both the TST and FST. Notably, these emotional abnormalities were not accompanied by changes in total locomotor activity or by deficits in spatial memory tasks. This double dissociation strongly supports the concept that ventral hippocampal connectivity with OFC is preferentially involved in affective regulation, whereas dorsal hippocampal connectivity with OFC is more relevant for spatial cognition. Such segregation parallels the broader functional dichotomy between dorsal and ventral hippocampal domains (Bannerman et al., 2014; Fanselow and Dong, 2010; Strange et al., 2014) and provides direct circuit-level evidence that the OFC interfaces with these domains in different ways.

These findings may also have broader translational relevance, as developmental alterations in large-scale cortical network organization and molecular vulnerabilities affecting neuronal circuit integrity have been implicated in cognitive and psychiatric dysfunction (Wu et al., 2025; Ran et al., 2026). Understanding how anatomically distinct OFC–hippocampal pathways contribute to emotional and cognitive regulation may therefore provide insight into mechanisms underlying vulnerability to neuropsychiatric disorders.

Several limitations should be considered when interpreting the present findings. Although the viral tracing strategy used here provides anatomical evidence for directional and polysynaptic connectivity, it does not fully define the synaptic and physiological properties of the identified pathways. In addition, the taCasp3-based disconnection approach establishes circuit-level necessity but does not resolve the relative contribution of individual relay nodes or projection-defined neuronal populations. Moreover, because the present study employed a crossed unilateral disconnection strategy rather than complete bilateral lesions of individual brain regions, the observed behavioral phenotypes should be interpreted as reflecting disruption of interregional functional communication rather than total loss of OFC or hippocampal function. Because these pathways involve polysynaptic relay structures rather than exclusively direct monosynaptic projections, the observed behavioral effects should be interpreted at the level of circuit interactions rather than isolated direct synaptic connectivity. Furthermore, the present disconnection strategy primarily addresses the necessity of these pathways but does not determine whether enhanced activity within OFC–hippocampal circuits would produce complementary behavioral effects. Future studies using bidirectional pathway-specific manipulations, such as optogenetic or chemogenetic activation approaches, will be necessary to further clarify the causal roles of these circuits. Finally, because only male mice were examined and the behavioral assessment focused primarily on spatial memory and affect-related phenotypes, caution is warranted when generalizing these findings across sexes and behavioral domains.

In conclusion, our study demonstrates that the OFC is linked to dorsal and ventral hippocampal CA1 subregions through anatomically distinct circuits with dissociable behavioral functions. These findings provide a circuit-level framework for understanding how the OFC interacts with longitudinally specialized hippocampal networks to differentially regulate spatial memory and affect-related behaviors, and offer insight into the organization of hippocampal–orbitofrontal communication.

## Data Availability

The original contributions presented in the study are included in the article/supplementary material, further inquiries can be directed to the corresponding author/s.
